# Supplementary figures and images for: The Establishment of Evaluation Models for the Cooking Suitability of Different Pork Muscles
Source: Foods. 2023 Feb 8;12(4):742. doi: 10.3390/foods12040742 (PMC9956234; doi:10.3390/foods12040742)

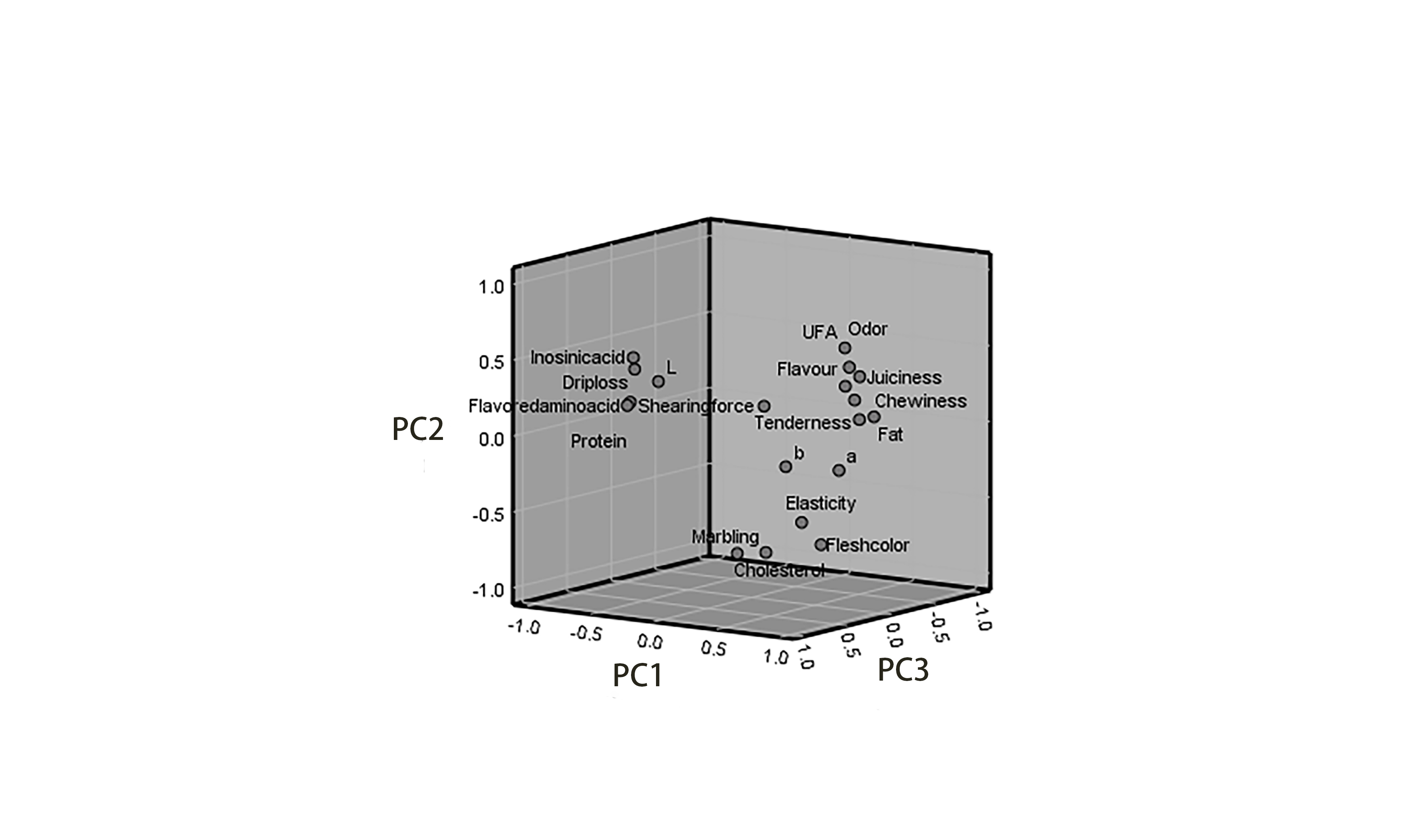

Supplement: Supplementary file 1 [file foods-12-00742-s001.zip › Supplementary File/Figure S1 PCA loading diagram under boiling method.png]

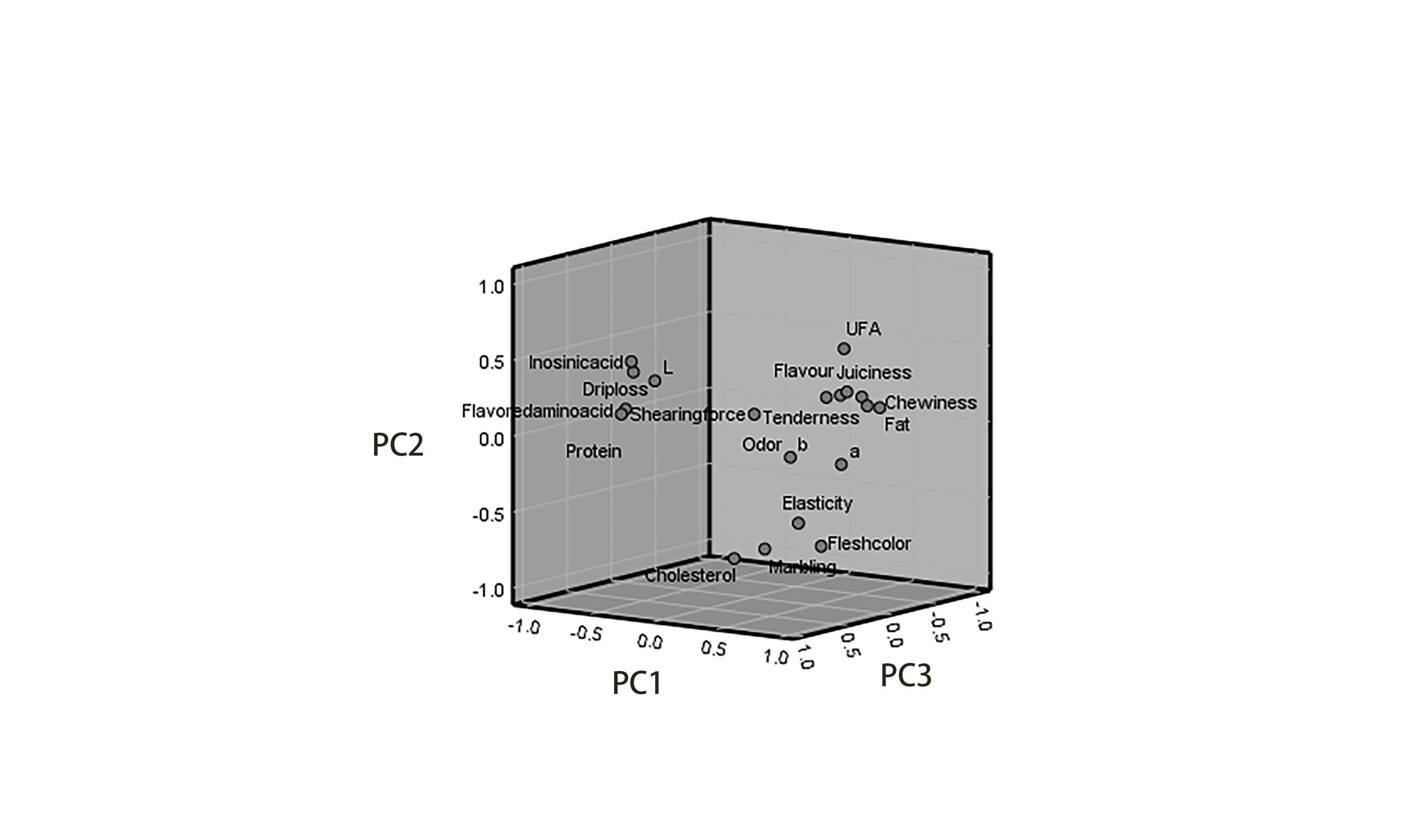

Supplement: Supplementary file 1 [file foods-12-00742-s001.zip › Supplementary File/Figure S2 PCA loading diagram under scalding method.png]

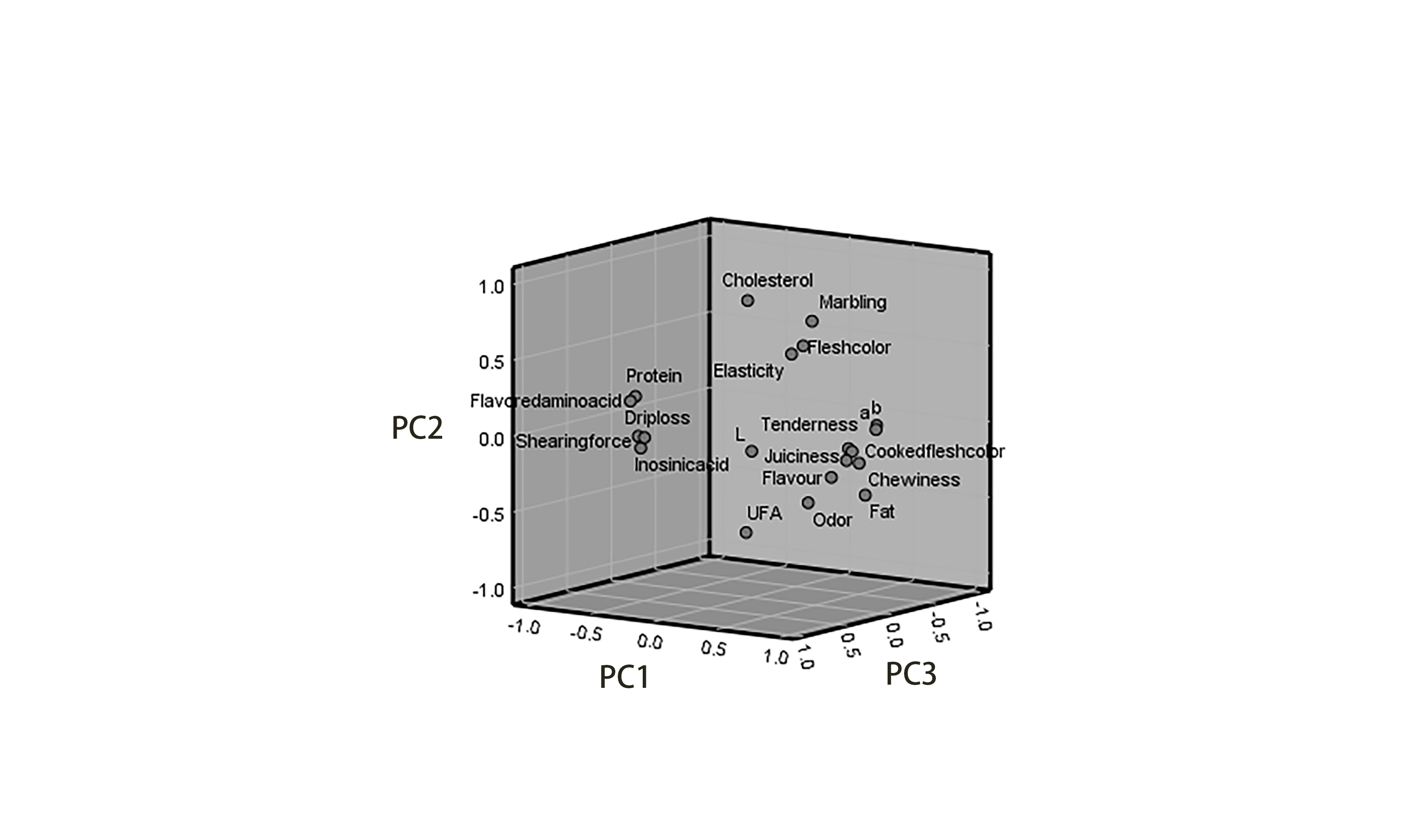

Supplement: Supplementary file 1 [file foods-12-00742-s001.zip › Supplementary File/Figure S3 PCA loading diagram under roasting method.png]
